# Evaluation of High-Dose Vitamin A Treatment in Postmolar Patients with Low and Plateauing Serum Human Chorionic Gonadotropin Levels*

**DOI:** 10.1055/s-0040-1710302

**Published:** 2020-05

**Authors:** Elza Maria Hartmann Uberti, Ruth Karina Escobar Diaz, Rodrigo Bernardes Cardoso, Antonio Braga

**Affiliations:** 1 Porto Alegre Trophoblastic Disease Center, Mario Totta Maternity Ward, Hospital Irmandade da Santa Casa de Misericórdia, Porto Alegre, RS, Brazil; 2 Rio de Janeiro Gestational Trophoblastic Disease Reference Center, Department of Obstetrics and Gynecology, Maternity School, Universidade Federal do Rio de Janeiro, Rio de Janeiro, RJ, Brazil

**Keywords:** gestational trophoblastic disease, low-and-plateauing serum hCG levels, high-dose vitamin A, doença trofoblástica gestacional, níveis de hCG sérico baixos e em “plateau”, alta dose de vitamina A

## Abstract

**Objective** To compare the effect of high-dose vitamin A (HD Vit-A) use during postmolar follow-up of patients with low and plateauing (L&P) serum human chorionic gonadotropin (hCG) levels, from the moment serum hCG plateaued (P-hCG) to the first normal serum hCG value (< 5 IU/L).

**Methods** The present retrospective series case study compared two nonconcurrent cohorts of patients. Control group (CG): 34 patients with L&P serum hCG levels who underwent expectant management for 6 months after uterine evacuation, from 1992 to 2010; study group (SG): 32 patients in similar conditions who received 200,000 IU of Vit-A daily, from the identification of a P-hCG level to the first normal hCG value or the diagnosis of progression to gestational trophoblastic neoplasia (GTN), from 2011 to 2017. The present study was approved by the Ethics Committee of the institution where it was conducted.

**Results** In both groups, the prevalence of persistent L&P serum hCG levels was < 5%. In the SG, hCG levels at plateau were higher (CG = 85.5 versus SG = 195 IU/L; *p* = 0.028), the rate of postmolar GTN was lower (CG = 29.4% versus SG = 6.3%, *p* = 0.034) and follow-up was shorter (CG = 14 versus SG = 10 months, *p* < 0.001). During GTN follow-up, there were no differences in GTN staging or treatment aggressiveness in both groups. High-dose Vit-A use did not have any relevant toxic effect. There were no GTN relapses or deaths.

**Conclusion** The limited use of HD Vit-A seems to have a safe and significant effect on the treatment of postmolar patients with L&P serum hCG levels and may decrease the development of postmolar GTN in this population.

## Resumo

**Objetivo** Comparar o efeito de alta dose de vitamina A (VitA) no seguimento pós-molar de pacientes com gonadotrofina coriônica humana (hCG) sérica apresentando valores baixos e em platô (L&P).

**Métodos** Estudo retrospectivo de série de casos comparando duas coortes não simultâneas. Grupo controle (CG): 34 pacientes com títulos de hCG sérico L&P submetidos a manejo expectante por 6 meses após o esvaziamento uterino, de 1992 a 2010; Grupo de Estudo (SG): de 2011 a 2017, 32 pacientes em condições semelhantes de hCG receberam Vit-A na dose de 200.000 IU por dia, do momento da identificação do hCG em platô ate o primeiro hCG normal ou diagnóstico de progressão para neoplasia trofoblástica gestacional (NTG). O presente estudo foi aprovado pelo Comitê de Ética da Instituição na qual foi desenvolvido.

**Resultados** Em ambos os grupos, a prevalência de hCG L&P foi < 5%. No SG, os níveis de hCG em platô foram maiores (CG = 85.5 *versus* SG = 195 IU/L; *p* = 0,028), e foram significantemente menores tanto a prevalência de NTG pós-molar (CG = 29.4% *versus* SG = 6.3%, *p* = 0,034) como o tempo de seguimento (CG = 14 *versus* SG = 10 meses, *p* < 0.001). Na evolução para NTG não houve diferença no estadiamento da International Federation of Gynecology and Obstetrics (FIGO, na sigla em inglês) ou na agressividade do tratamento. Com altas doses de Vit-A não houve qualquer efeito tóxico relevante. Não houve casos de recidiva de NTG ou de óbito.

**Conclusão** O uso limitado de altas doses de Vit-A parace ser seguro e apresenta efeitos significativos na evolução de pacientes em controle pós-molar com títulos de hCG sérico L&P, e pode diminuir o desenvolvimento de NTG pós-molar nessa população.

## Introduction

Gestational trophoblastic diseases (GTDs) comprise a spectrum of occasional disorders on human trophoblast derived from accidents occurred in the moment of fertilization. Gestational trophoblastic diseases include six clinical and pathological entities with different rates of remission, invasion and metastatic spread, from benign forms, such as the complete and partial hydatidiform moles (HM), to malignant forms, classified as gestational trophoblastic neoplasia (GTN), which include invasive HM, choriocarcinoma, placental site trophoblastic tumor and epithelioid trophoblastic tumor.^1^ ^2^ ^3^ ^4^

Human chorionic gonadotropin (hCG), a hormone produced by the placenta in any type of pregnancy, is the biologic tumor marker of GTD. Quantitative serum hCG testing, an aid in the diagnosis of HM and the pillar of postmolar follow-up, provides information that may be used to predict cases that will go into spontaneous remission or, on the contrary, that will develop GTN. The same information may be useful to control response to chemotherapy (ChT) and to surgery, as well as to detect rare cases of relapse.^5^ ^6^

Estimates of HM incidence indicate that it is 5 to 10 times more frequent in Brazil than in the United States and Europe.^7^ ^8^ ^9^ ^10^ Epidemiological studies suggest that genetic, immunological and diet variations may be responsible for this higher incidence.^2^ ^5^ ^11^ Some studies found that a low level of vitamin A (Vit-A) in the diet may be associated with a higher risk of HM incidence, as well as with its progression into GTN.^11^ ^12^ ^13^ ^14^

During postmolar follow-up, persistence of low and plateauing (L&P) serum hCG levels is not frequent, as it affects < 5% of the patients.^15^ ^16^ Although this condition may increase the emotional stress of HM for these women, few treatment options are available, which may lead to a decision to initiate ChT immediately to ensure cure.^15^ ^16^ Although the International Federation of Gynecology and Obstetrics (FIGO) recommended, until 2018, ChT initiation 6 months after uterine evacuation for patients with L&P serum hCG levels, some options have been put forth to avoid immediate ChT initiation, such as prolonged hCG surveillance, repeat curettage, or treatment with high doses of Vit-A.^17^ ^18^ ^19^ ^20^ ^21^ ^22^

In vitro and in vivo studies have demonstrated the beneficial effects of the administration of high doses (HD) of Vit-A in cases of GTD. Chiu et al^21^ studied choriocarcinoma cells in vitro and found that all-trans retinoic acid, a Vit-A derivative, inhibited cell invasion and proliferation and induced apoptosis. Andrijono et al^22^ concluded that the increased apoptotic activity of trophoblastic cells was proportional to the amount of retinoic acid used. The recommended dose of Vit-A is up to 10,000 IU/daily; however, in studies including GTD patients, high-dose vitamin A (HD Vit-A) (200,000 IU/daily) was administered to prevent postmolar GTN.^14^ In 2010, Andrijono et al^14^ published a clinical trial that found a significant reduction in the incidence of postmolar GTN in patients treated with HD Vit-A (200,000 IU/daily) administered from uterine evacuation to HM remission, with no GTD-associated morbidity.

The present study evaluated the same HD Vit-A use in a group of patients with L&P serum hCG levels during postmolar follow-up. The identification of alternative treatments for these cases of GTD may prevent the exposure of young women to chemotherapeutic agents and their deleterious immediate and future adverse events.

## Methods

This retrospective case series compared clinical outcomes of patients with HM and L&P serum hCG levels followed-up in two independent cohorts by the same multidisciplinary team at the GTD Reference Center (RC) of the Irmandade da Santa Casa de Misericórdia Hospital (ISCMPA, in the Portuguese acronym), Porto Alegre, RS, Brazil. Control group (CG): of the 1,370 patients with HM followed-up between January 1997 and December 2010 in the RC, 34 (2.5%) had L&P serum hCG levels and underwent expectant management for at least 6 months after uterine evacuation.

Study group (SG): of the 734 patients with HM followed up in the RC from January 2011 to December 2017, 32 (4.3%) had L&P serum hCG levels and were administered Vit-A 200,000 IU/daily from L&P hCG identification to the main outcome (spontaneous remission of the disease or postmolar GTN)

After uterine evacuation of molar pregnancy, all patients underwent clinical and laboratory follow-up at 7–14 days intervals with quantitative serum hCG testing using an electrochemiluminescent immunoassay (DPC Immulite, Siemens, Los Angeles, CA, USA) until remission of the disease.^23^ After that, testing continued monthly for at least 6 more months, in case of maintained spontaneous remission of the disease, or for 12 months, in cases of remission after treatment of GTN. The patients were then discharged from postmolar follow-up and were told they might try to become pregnant again. At each visit, from uterine evacuation to the completion of follow-up, all patients received instructions about the use of contraceptives, and they were systematically asked about that use.^24^ Gestational trophoblastic disease remission was defined as three consecutive weekly serum hCG measurements < 5 IU/L.^6^ Patients were diagnosed with L&P serum hCG levels when the results of four consecutive weekly serum hCG measurements had a variation of ∼ 10% and were all < 1,000 IU/L.^15^ ^16^ ^25^ ^26^ ^27^

The diagnostic criteria of GTN for prompt ChT initiation were those established by the FIGO 2000 guidelines:^28^

a) Four or more plateauing serum hCG results over 3 weeks, that is, on days 1, 7, 14 and 21;
b) Increase of serum hCG levels for ≥ 3 consecutive measurements for at least 2 weeks; that is, on days 1, 7 and 14;
c) Histological diagnosis of choriocarcinoma;
d) Elevated hCG levels for 6 months or longer after uterine evacuation.

Patients with L&P serum hCG levels first underwent pelvic examination, pelvic transvaginal Doppler sonography and chest X-ray to investigate the possible presence of invasive HM or metastatic disease. In case the chest X-ray was not conclusive, or if there were metastases > 1.00 cm, chest CT and brain MRI scans were used to magnify the screening for metastases.^28^

If no uterine or metastatic lesions were detected during the postmolar follow-up of patients with L&P serum hCG levels, those in the CG were only followed-up closely with serum hCG measurements every 15 days until the outcome, or for 6 months after uterine evacuation. After that time, if levels remained > 5 IU/L, the fourth FIGO criterion for ChT initiation was confirmed.^28^ For patients in the SG, HD Vit-A 200,000 IU/daily tablets PO was indicated after informed consent and was administered from the plateau diagnosis to the main outcome, for ∼ between 30 and 60 days. At each 15 days appointment of the postmolar follow-up, the SG patients were asked if they had adverse events of HD Vit-A and, associated with hCG measurements, also had control of hepatic toxicity with hepatic enzyme assessment during and 1 month after HD Vit-A use. The primary outcome was spontaneous remission or progression to GTN in both groups, according to the FIGO criteria. Secondary outcomes were the evaluation of adverse events in patients treated with HD Vit-A and, in the two groups, time to remission of the disease, clinical features of subsequent GTN, relapse and death. The epidemiological variables under study in the two groups were: age, parity, gestational age at time of uterine evacuation, HM histology, serum hCG level at time of plateauing, time from plateau diagnosis to first normal hCG result (< 5 IU/L), spontaneous disease remission or progression to GTN, clinical characteristics of subsequent GTN, GTD relapse and death. For patients in the SG, the following variables were also studied: time from HD Vit-A initiation and outcomes, and adverse events associated with HD Vit-A use.

Analyses were made using absolute frequencies and percentages, and results were compared using the Student *t*, the Mann-Whitney and the Pearson chi-squared tests. The level of significance was set at α < 0.05. The present study was approved by the Ethics Committee of the ISCMPA under the number 1.662.695.

## Results

**Fig. 1:**
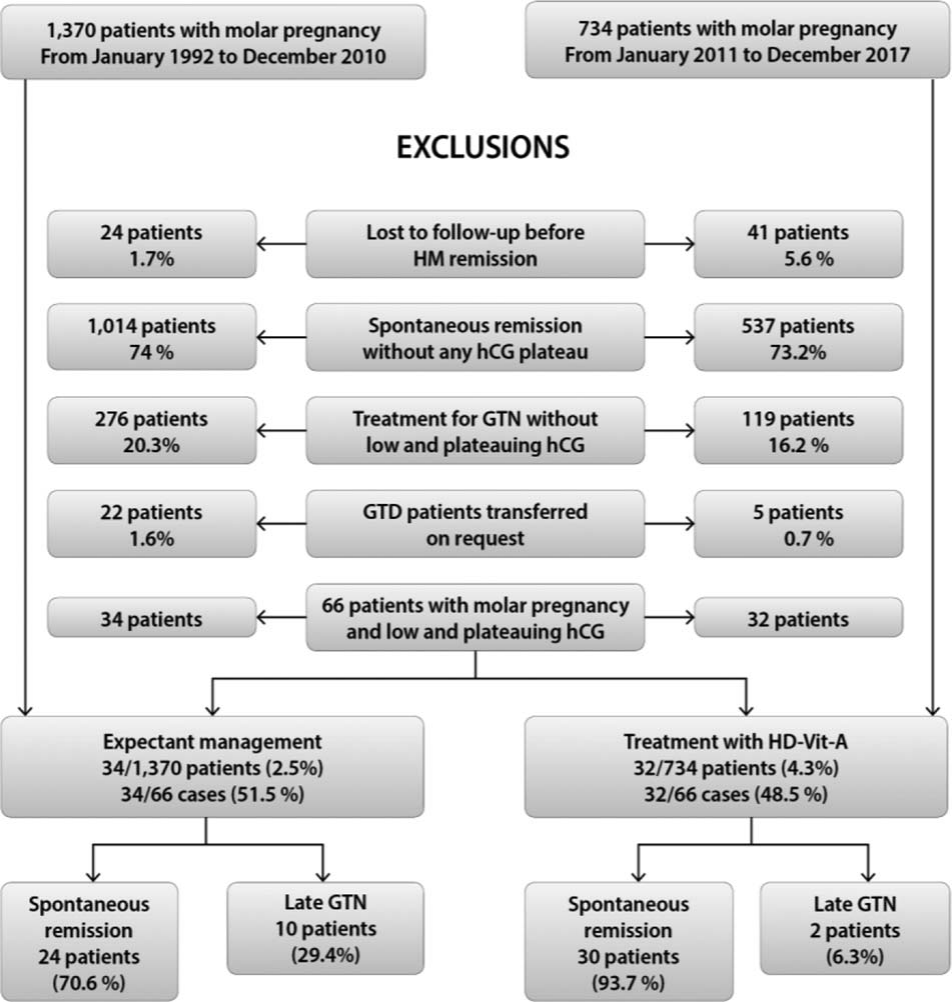
Flow diagram summarizing the derivation of the study population.

Fig. 1 is a flow diagram of the derivation of the patient population, with outcomes according to the different types of treatment. The prevalence of patients with L&P serum hCG levels undergoing postmolar follow-up was < 5%.

Table 1 shows the clinical variables for patients with L&P serum hCG levels according to expectant management or use of HD Vit-A.

**Table 1:** Clinical variables for patients with low and plateauing serum human chorionic gonadotropin levels according to expectant management or use of high-dose vitamin A

Variables	Expectant management (*n* = 34)	Vitamin A use (*n* = 32)	*p-value*
Age, years old^1^	31.4 ± 9.7 [16–52]	29.5 ± 7.5 [15–44]	0.369^t^
Number of gestations^2^	1 (1 - 2)	1 (1 - 2)	0.89^M^
Parity^2^	0 (0 - 1)	1 (0 - 2)	0.065^M^
Pre-evacuation hCG level, IU/L^2^	40,600 (285–195,779)	85,854 (741–166,890)	0.829^P^
Gestational age at diagnosis, weeks^1^	11.9 ± 3.8 (5–22)	11.8 ± 4.7 (5–29)	0.947^t^
HM histology – N (%)^3^			0.479^M^
* Complete hydatidiform mole*	21 (63.6)	21 (67.7)	
* Partial hydatidiform mole*	12 (36.4)	10 (34.5)	
hCG level at plateau, IU/L^2^	82.5 (35–207)	195 (61–664)	0.028^P^
Interval between uterine evacuation and hCG plateau, weeks	12.7 ± 5.8 [8–38]	9.8 ± 3.4 [5–17]	0.019^t^
Interval between hCG plateau and outcome, days	60 (39–91) [9–160]	50 (35–70) [16–290]	0.374^M^
Duration of vitamin A treatment, days^2^	–	40 (30–60)	–
Time to hCG normalization, days^2^	65 (44–95)	44 (30–71)	0.327^P^
Duration of follow-up, months^2^	14 (11–18)	10 (8–11)	< 0.001^P^
Required chemotherapy due to GTN – N (%)	10/34 (29.4%)	2/32 (6.3%)	0.034^M^

Abbreviations:GTN, gestational trophoblastic neoplasia; hCG, human chorionic gonadotropin; HM, hydatidiform mole.

1. Mean (Standard derivation, range); 2. Median (interquartile); 3. No histological differentiation between complete or partial mole occurred in two cases; t, Student *t* test.; M, Mann-Whitney test.; P, Pearson chi-squared test.

**Fig. 2:**
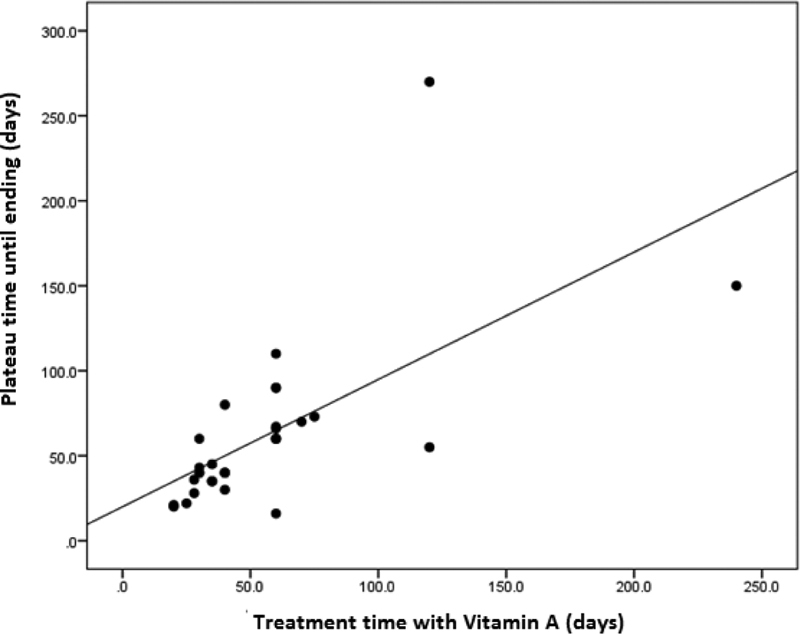
Duration of vitamin A use according to time from plateau to outcome (days).

Mean age (CG = 31.4 ± 9.7 versus SG = 29.5 ± 7.5 years old; *p* = 0.369), median hCG serum level before uterine evacuation (CG = 40,600 versus SG = 85,854 IU/L; *p* = 0.829), mean gestational age at HM diagnosis (CG = 11.9 ± 3.8 versus SG = 11.8 ± 4.7 weeks; *p* = 0.947) and HM histology (*p* = 0.479) were similar in both groups. At the time of hCG plateau, the median hCG level was significantly higher in the SG (82.5 versus 195 IU/L; *p* = 0.028), as well as the frequency of serum hCG levels > 300 IU/L (CG =17.6% versus SG = 46.9%; *p* = 0.014). In the group of patients treated with HD Vit-A, the occurrence of postmolar GTN was 6.3%, significantly lower than that found in the group of those that underwent expectant management (29.4%, *p* = 0.034). The duration of postmolar follow-up was significantly shorter in the SG (CG = 14 months versus SG = 10 months, *p* < 0.001). The median duration of HD Vit-A use was 40 (30–60) days, and there were no differences in frequency of effect when the duration was ≥ 30 days, as shown in Fig. 2.

Table 2 compares the variables for patients treated for GTN after expectant management or HD Vit-A use.

**Table 2:** Treatment and outcomes of women with gestational trophoblastic neoplasia and persistent low and plateauing serum human chorionic gonadotropin levels after postmolar follow-up according to expectant management or high-dose vitamin A use

Variable	ChT after expectant management N = 10	ChT after high-dose Vitamin A use *n* = 02	*p-value*
Age, years old, mean ± SD,^1^ [min - max]	33.8 ± 9.5 (25–51)	22.5 ± 2.1 (21–24)	0.136^t^
Evacuation – N (%)			1.000^P^
* In the RC*	7 (70)	2 (100)	
* Out of the RC*	3 (30)	0	
Pre-evacuation hCG level, IU/L, median ± IQA^2^	556.000 (369–1.317.065)	283.663 (193.755–373.571)	0.909^M^
Gestational age at diagnosis, weeks, mean ± SD,^1^ [min - max]	13.1 ± 1.5 (11–16)	10 ± 0 (10–10)	0.017^t^
HM^c^ histology – N (%)			1.000^P^
* Complete hydatidiform mole*	9 (90)	2 (100)	
* Partial hydatidiform mole*	1 (10)	0 (0)	
hCG level at plateau, IU/L, median ± IQA^2^	82.5 (43–409)	658.5 (654–663)	0.030^M^
Time from evacuation to ChT initiation, weeks, median ± IQA^2^	25 (13.8–28)	16 (16–16)	0.727^M^
Pretreatment hCG level, IU/L, median ± IQA^2^	90.5 (53.3–314)	3.231 (567–3.231)	0.182^M^
Occurrence of metastasis – N (%)			1.000^P^
* No*	10 (100)	2 (100)	
* Yes*	0	0	
WHO/FIGO prognostic risk score – N (%)			1.000^P^
* Low risk*	10 (100)	2 (100)	
* High risk*	0	0	
Treatment – N (%)			1.000^P^
* Clinical, single-agent ChT*	8 (80)	2 (100)	
* Clinical, multi-agent ChT*	0	0	
* Hysterectomy + single-agent ChT*	2 (20)	0	
Number of ChT cycles to remission, mean ± SD,^1^ [min–max]	1.8 ± 1.3 (1–5)	2.5 ± 0.5 (2–3)	0.407^t^
Lenght of follow-up, months, median ± IQA^2^	48 (15–67)	39.5 (35–44)	1.000^M^
Occurrence of relapse or death – N (%)	0	0	–

Abbreviations: ChT; Chemotherapy; hCG, human chorionic gonadotropin; HM; Hydatidiform mole; RC, referral center; SD, standard deviation; WHO/FIGO,. World Health Organization/ International Federation of Gynecology and Obstetrics.

1. Mean (Standard deviation, range); 2. Median (interquartile); t. Student *t* test; P. Pearson chi-squared test; M. Mann-Whitney test.

The following variables were similar: mean age, place where uterine evacuation was performed, serum hCG values before uterine evacuation, HM histology, time between molar evacuation and ChT initiation, serum hCG values before ChT, occurrence of metastases, World Health Organization (WHO)/FIGO prognostic risk score, type of treatment, number of ChT cycles until GTN remission, and duration of follow-up. Among patients with persistent L&P serum hCG levels initially treated with Vit-A, serum hCG levels were significantly higher than those in the group that received expectant management (CG = 82.5 IU/L versus SG = 658.5 UI/L; *p* = 0.030).

In the group treated with HD Vit-A, no patient had hepatic toxicity according to serum aspartate aminotransferase levels; only one (3.3%) patient had a mild side effect, dry skin, being resolved after the drug was discontinued. In both groups, adherence to follow-up was good; most patients received a medical discharge after postmolar follow-up was complete (CG = 88.2% versus SG = 90.6%; *p* = 0.553). There were no cases of relapse or death of patients followed-up in the present study.

## Discussion

To our knowledge, no study has evaluated the use of HD Vit-A in the postmolar follow-up of patients with persistent L&P serum hCG levels, as we did in this nonconcurrent retrospective cohort. Even though serum hCG levels were higher in the SG, the administration of limited HD Vit-A daily reduced significantly the development of postmolar GTN and the length of follow-up. Clinically (external validity), our treatment of women with a molar pregnancy and persistent L&P serum hCG levels using HD Vit-A resulted in a 3-fold increase in the number of cases of spontaneous remission, a finding that is in agreement with that reported by Andrijono et al.^14^ However, those authors used HD Vit-A for patients with complete HM since uterine evacuation to GTD outcome, which led to a measurement bias in their results, as they did not include the high percentage of expected spontaneous remission (∼ 80%), as well as the possible risks of HD Vit-A supplementation for long periods.^1^ ^2^ ^3^ ^4^ ^5^ ^6^ ^7^

For patients initially treated with HD Vit-A in our study, even in cases that later required ChT, the prognosis of treatment outcome was similar to that of patients that underwent initially expectant management after the diagnosis of persistent L&P serum hCG levels. This may be assigned to the short duration of HD Vit-A use (median of 40 days), which did not have any significantly important effects on time to ChT initiation, WHO/FIGO prognostic risk score, or even response to ChT regimens in the group of patients that progressed to GTN when compared with those patients that underwent expectant management initially.

Treatment with HD Vit-A has been associated with inhibition of the development of experimental tumors of the skin, breast, mouth, ovaries, lung, liver, gastrointestinal tract, prostrate and urinary bladder.^29^ ^30^ ^31^ All types of trophoblastic cells in the placenta, such as syncytiotrophoblasts, cytotrophoblasts and intermediate trophoblasts, have receptors for retinoic acid (RA), the final product of Vit-A metabolism in the organism. As RA binds to retinol binding protein (RBP) receptors, the complex RA-receptor acts in the control of cell proliferation, inducing trophoblastic apoptosis by stimulation of apaf-1, caspase-7, caspase-9 and dab-2, and inhibition of Bcl-2.^22^ According to Andrijono et al,^22^ the administration of RA to trophoblastic cells increased their apoptotic activity and interrupted the cell cycle at the G1 and S phases.^32^

The lowest effective dose of HD Vit-A to achieve remission of patients with molar pregnancies and persistent L&P serum hCG levels, or in the associated treatment of GTN, has not been determined.^33^ ^34^ The dose used in our study was the same suggested by Andrijono et al,^14^ but we administered it for a shorter time, from the detection of persistent L&P serum hCG levels to the outcome. The HD Vit-A dose in our study, 200,000 IU/day, did not have any important adverse effects, evaluated at each follow-up visit. No patient had any changes in hepatic function during follow-up, and only one patient had a mild adverse effect, dry skin, which resolved after Vit-A discontinuation. These data are in agreement with those reported by Andrijono et al.^14^ The use of HD Vit-A for > 30 days did not improve prognoses, which suggests, therefore, that there is an optimal period after which it would not be necessary to continue the treatment.

One of the important limitations of our study is that, although data were collected from a single GTD RC in Southern Brazil, where patient compliance is high because patients have been followed-up by the same professional (one of the authors) since 1985, findings may not reflect the reality of other Brazilian RCs or the general population. The reduced number of patients that progressed to GTN in both series has also limited the analyses of our results. The most important study limitation, however, was its design, as retrospective analyses have biases that may limit their generalization.

## Conclusion

In the present study, postmolar GTN was less frequent and the length of follow-up was shorter in the group of women with a molar pregnancy and L&P serum hCG levels who received treatment with HD Vit-A. Vitamin A use was well tolerated in the population under study, and there were very few side effects, which resolved after treatment discontinuation. When HD Vit-A treatment failed and patients progressed to postmolar GTN, prognoses were similar to those of women treated with ChT after initially expectant management. Randomized multicenter studies with a higher impact should be conducted to confirm our findings about the beneficial effects and safety of HD Vit-A use to treat women with molar pregnancies and persistent L&P serum hCG levels.
